# A Case of Follicular Tumor of Uncertain Malignant Potential (FT-UMP) with Glomeruloid Features Showing Capsular Mucinous Degeneration

**DOI:** 10.1155/2021/1686025

**Published:** 2021-03-11

**Authors:** Daniela Cabibi, Andrea Mondello, Ada Maria Florena, Giulia Rimi, Antonino Giulio Giannone, Calogero Cipolla, Maria Rosaria Valerio, Giuseppa Graceffa

**Affiliations:** ^1^Department of Sciences for the Promotion of Health and Mother and Child Care, Anatomic Pathology, University of Palermo, Via Giovanni Pascoli, 6, 90133 Palermo, Italy; ^2^Department of Surgical, Oncological and Oral Sciences, Via del Vespro 129, 90127 Palermo, Italy

## Abstract

The most recent revision of the *World Health Organization (WHO) Classification of Tumours of Endocrine Organs* introduced a new variant of follicular thyroid carcinoma (FTC). It is characterized by a “glomeruloid” architectural pattern of growth. We present a case of follicular tumor with glomeruloid features, with Alcian Blue positive mucinous stromal degeneration in foci of questionable capsular microinvasion. At our knowledge, this the second case of glomeruloid follicular tumor in the literature and the first case in which Alcian Blue staining was used to investigate capsular invasion. Moreover, RAS mutation further supports that this is a variant of follicular tumor with uncertain malignant potential.

## 1. Introduction

The latest revision of the *World Health Organization Classification of Tumours of Endocrine Organs* introduced a new variant of follicular thyroid carcinoma (FTC) which is characterized by a “glomeruloid” architectural pattern of growth. The term *glomeruloid* refers to an architectural model that mimics the renal glomerulus, where the follicles contain round or oval epithelial tufts growing within them [1]. This pattern has been previously reported in some tumors, such as prostate adenocarcinoma, breast carcinoma, mesothelioma, and vascular tumors [2, 3]. To date, only one case of glomeruloid FTC has been reported in medical literature upon thyroid carcinomas [4].

Given the rarity of this neoplasia and difficulty of the diagnosis, we report a new case of follicular tumor with glomeruloid features, in which the diagnosis of malignancy was made more difficult by very focal capsular invasion. However, the presence of mucinous stromal degeneration, better highlighted by Alcian Blue staining, was useful to detect it. To our knowledge, this is the second case of follicular thyroid carcinoma with glomeruloid features reported in the literature and the first thyroid neoplasia with focal capsular invasion highlighted by Alcian Blue mucinous stromal degeneration.

## 2. Case Presentation

A 40-year-old woman with history of Hashimoto thyroiditis came to our hospital after an endocrinologist discovered a palpable mass in the neck, on the left. The patient had no compressive symptoms. The TSH level was normal at 1.12 mIU/L. Physical examination revealed an enlarged thyroid with a 30 mm thyroid nodule with smooth edges. Thyroid sonography showed an oval hypoechoic nodule in the left lobe of the thyroid, surrounded by a thin, regular hyperechoic border, measuring 32 mm on its maximum diameter; the rest of the gland was slightly enlarged. FNA biopsy was performed. The cytologic diagnosis was “follicular hyperplastic nodule” (Tir 2 SIAPEC-IAP 2014; Cat. II Bethesda 2008). Despite the cytological diagnosis, because of the progressive enlargement of the nodule, a total thyroidectomy was performed.

### 2.1. Pathologic Findings

Grossly, the nodule was located in the upper and middle third of the left lobe of the thyroid gland. It measured 2.5 × 2 × 1.4 cm and appeared solid, encapsulated, well-defined, and orange-greyish with a central hemorrhagic area. The surgical specimen was fixed in neutral, buffered 10% formalin, and paraffin-embedded sections were stained with hematoxylin and eosin and with Alcian Blue staining. The immunohistochemical staining was carried out with the BenchMark XT automated slide staining system (Ventana Medical Systems, Tucson, AZ) according to the manufacturer's instructions, by using the following antibodies: TTF1, galectin-3 (Gal-3), CK19, HBME-1, 34betaE12, and p53. Ki67.

As control, we retrieved from the archives of our institution fifteen cases of minimally invasive follicular carcinoma consecutively observed in the last five years and fifteen cases of follicular adenoma consecutively observed in the last two years. We stained them with Alcian Blue staining.

Genomic DNA was extracted from 10 *μ*m paraffin-embedded tumor sections. Slides were microscopically examined, and tumor areas were marked and carefully dissected under microscopic observation. Dissected material was deparaffinized in xylene, washed in ethanol, and rehydrated. DNA extraction was performed using the QIAamp Tissue Kit (Qiagen, Hilden, Germany) according to the manufacturer's protocol.

**Figure 1:**
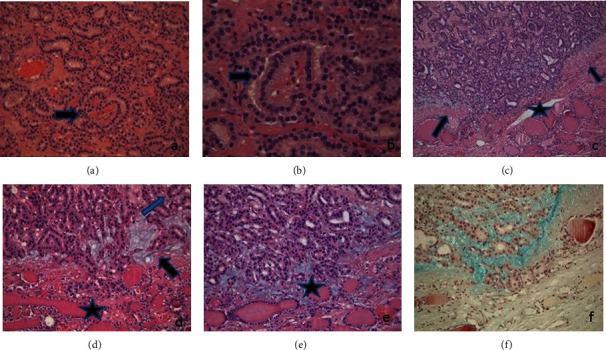
Neoplasia with microfollicular pattern of growth. The follicles are lined by cuboidal cells, lacking nuclear crowding and overlapping. The arrows mark rare epithelial tufts growing within the follicles, with a fibrovascular core, mimicking the appearance of renal glomerulus (a, b) and foci of capsular microinvasion (c, d, e) with stromal mucinous material (arrows) staining positively with Alcian Blue staining (f). H-E staining: (a) =200x, (b) =400x, (c) =100x, (d) =200x, (e) =200x, and (f) = Alcian Blue staining, 200x.

The histologic examination of the nodule showed a partially encapsulated lesion with a microfollicular pattern of growth. The follicles were often empty of colloid and lined by cuboidal cells, without nuclear crowding and pseudostratification. The presence of epithelial tufts growing within the follicles, sometimes with a fibrovascular core, thus mimicking the appearance of renal glomerulus, was occasionally detected (Figures 1(a) and 1(b)).

Nuclei were small, round, and oval, with evenly distributed chromatin and absent or, rarely, inconspicuous nucleoli. Nuclear features of papillary carcinoma (i.e., grooves, optically clear nuclei, or intranuclear cytoplasmic inclusions) were not detected.

**Figure 2:**
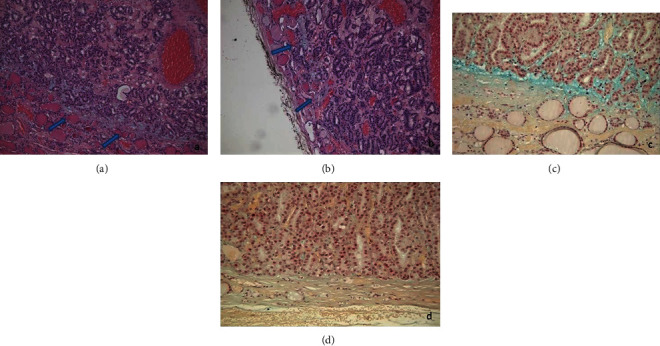
Stromal mucinous material (a, b) positive for Alcian Blue staining (c) at the interface between the tumor and the surrounding parenchyma, in areas in which the capsule was absent (arrows). Stromal mucinous material was absent where the capsule was intact (d). (a, b) H-E staining, 200x. (c, d) Alcian blue staining, 200x.

The capsule was thin, and after careful searching, a few foci of questionable capsular microinvasion were found. Interestingly, mucinous stromal material was evidenced at the periphery of the lesion, in foci of microinvasion (Figures 1(c)–(e)) and at the interface between the tumor and the surrounding parenchyma, where the capsule was absent (Figures 2(a) and 2(b)). Mucinous stromal material stained positively with Alcian Blue staining (Figures 1(f) and 2(c)). It was absent where the capsule was intact (Figure 2(d)) and in the central areas of the lesion (Figure 1). The immunohistochemical assay showed positive immunostaining for TTF1 and negative immunostaining for HBME-1, Gal-3, 34betaE12, CK19, and p53. Ki67 stained positively in 5% of nuclei. Alcian Blue positive material was not seen either in the foci of microinvasion of the follicular tumor or near the capsule of follicular adenomas used as control. Cytogenetic analysis showed a mutation in exon 2 of N-RAS. The mutation in codon 61 consisted of a CAA to CGA change that leads to a substitution of glutamine by an arginine (p.Q61R), detected by a real-time, allele-specific amplification essentially as described by Castro et al. [5].

## 3. Discussion

Follicular thyroid neoplasia with glomeruloid features is very rare and since the first report, [4] no other cases have been reported in literature except one case of follicular glomeruloid adenoma [6].

It is important to be aware of the existence of this neoplasia because the glomeruloid bodies could architecturally simulate the papillae of papillary carcinoma, from whom they differ due to the lack of typical nuclear features. On the other hand, because of the bland nuclear features, glomeruloid bodies may be underestimated, mainly when they are scanty. Therefore, some cases of glomeruloid carcinoma could be underdiagnosed.

In our case, according to previous reports, the absence of true papillae, of papillary nuclear changes, and the negative immunostaining for HBME-1, Galectine 3, and CK19 suggests the diagnosis of follicular neoplasia, in keeping with cytogenetic analysis, showing N-RAS mutation [7]. In contrast with the first case reported in the literature [4], some differences were evidenced: in our case, the follicles were made of bland cuboidal cells with no overlapping, unlike tall and sometimes overlapping columnar cells described by Cameselle-Teijeiro et al. [4]; the tumor showed only minimal capsular invasion and stained negatively for 34betaE12, CK19, p53, and HBME-1. The bland cytological features, the absence of evident capsular invasion, and the immunohistochemical pattern made the diagnosis of malignancy even more difficult. We would emphasize the presence of Alcian blue positive stromal mucinous material near the foci of questionable capsular microinvasion. Where the capsule was absent, stromal mucinous degeneration was present at the interface between the tumor and the surrounding parenchyma. No extracellular mucin deposition was found in the central areas of the proliferation, unlike the extensive, random mucin deposition reported in some follicular adenomas [8]. So, this feature might evidence the advancing front of the neoplasia. We want to emphasize the Alcian Blue staining usefulness to highlight these foci of capsular microinvasion. Noteworthy, Alcian blue positive stromal mucinous degeneration was absent where the capsule was intact. None of the FTC used as controls showed mucinous Alcian Blue positive material in the foci of invasion, and neither it has been reported in previous literature. We could hypothesize that some enzymes secreted by the tumor, such as some metalloproteases, could induce stromal degradation, thus facilitating the invasion [9–11]. This hypothesis needs to be further investigated on a larger number of cases, even to confirm if this is a typical feature of “glomeruloid FTC.”

In keeping with the abovedescribed features, we diagnosed this case as “follicular tumor of uncertain malignant potential (FT-UMP) with glomeruloid features.”

The prognosis of the patient was excellent after four years follow-up, as expected for this diagnosis, but a longer follow-up is necessary.

This is the first case of follicular tumor of uncertain malignant potential (FT-UMP) with glomeruloid features showing mucinous Alcian Blue positive stromal degeneration in the foci of questionable capsular microinvasion. The evidence of RAS mutation has further supported the current WHO classification in considering this tumor as a form of follicular neoplasm.
